# An efficient and versatile reverse genetics system for bovine coronavirus facilitates antiviral evaluation

**DOI:** 10.1186/s13567-026-01851-6

**Published:** 2026-10-11

**Authors:** Yiran Zeng, Hanqiong Gao, Yuanlu Lu, Menglu Fan, Zhiyuan Liu, Yue Li, Linjuan Wu, Jun Xia, Jihui Ping

**Affiliations:** 1 Jiangsu Engineering Center of Animal Immunology and MOE International Joint Collaborative Research Laboratory for Animal Health and Food Safety, College of Veterinary Medicine, Nanjing Agricultural University, Nanjing, 210095 China; 2 College of Veterinary Medicine, Shangqiu Polytechnic, Shangqiu, 476000 China; 3 Xinjiang Academy of Animal Sciences Institute of Veterinary Medicine, Key Laboratory for Prevention and Control of Herbivorous Animal Diseases of the Ministry of Agriculture and Rural Affairs and Xinjiang Animal Disease Research Key Laboratory, Urumchi, 830000 China

**Keywords:** Bovine coronavirus, reverse genetics, CPER, reporter virus, antiviral evaluation

## Abstract

Bovine coronavirus (BCoV), a primary etiological agent of calf diarrhea, exhibits dual tropism for the respiratory and enteric tracts. However, research on BCoV gene function and pathogenesis has been limited by the lack of convenient and flexible reverse genetics tools. Herein, we report a robust reverse genetics system for BCoV based on an optimized circular polymerase extension reaction (CPER) methodology. We successfully rescued recombinant BCoV (rBCoV) in HRT-18G cells and engineered recombinant BCoV expressing ZsGreen fluorescent protein (rBCoV/Zs-T2A-ns2). Phenotypic analysis demonstrated that both unmodified rBCoV and rBCoV/Zs-T2A-ns2 maintained growth kinetics comparable to the wild-type BCoV. Importantly, the reporter virus demonstrated remarkable genetic stability for over ten passages, identifying the ns2 locus as a suitable insertion site for stable foreign gene expression without compromising viral fitness or sequence integrity. As a proof-of-concept application, the rBCoV/Zs-T2A-ns2 was employed for antiviral compound evaluation, successfully identifying four compounds—camostat, nafamostat, niclosamide, and nelfinavir—capable of effectively inhibiting BCoV replication. Further time-of-addition assays revealed that nelfinavir and niclosamide targeted the BCoV replication stage, while camostat and nafamostat interfered with BCoV internalization. This versatile CPER-based reverse genetics system provides a critical platform for advanced BCoV pathogenesis research and accelerated development of novel vaccines and effective antiviral therapeutics, ultimately contributing to enhanced bovine health and sustainable livestock production.

**Supplementary Information:**

The online version contains supplementary material available at https://doi.org/10.1186/s13567-026-01851-6.

## Introduction

Bovine coronavirus (BCoV), a member of the genus *Betacoronavirus*, is a pathogen of significant veterinary and economic importance, causing respiratory and enteric diseases in cattle worldwide [1]. BCoV has a broad host range, with RNA having been detected in cattle, yaks, musk deer, Daurian ground squirrels, goats, and camels [2–6]. Like other coronaviruses, including human betacoronaviruses such as HCoV-OC43, HCoV-HKU1, and SARS-CoV-2, BCoV possesses a large, positive-sense, single-stranded RNA genome of approximately 30 kilobases (kb). Approximately two-thirds of the viral genome at the 5′ end consists of a large replicase gene *ORF1ab*, which is proteolytically processed into 16 nonstructural proteins. The remaining third primarily encodes five structural proteins: hemagglutinin esterase (HE), spike protein (S), envelope protein (E), membrane protein (M), and nucleocapsid protein (N), as well as several accessory proteins (ns2, ns4.9, ns4.8, ns12.7) that modulate the host immune responses and are critical determinants of pathogenesis [7–10]. Among these, the ns2 protein, located in the cytoplasm, has been shown to antagonize the host interferon response, marking it as a key factor in viral virulence and host interaction [11–13].

Advances in viral reverse genetics have provided crucial technical support for investigating viral genome functions and clarifying viral infection mechanisms, serving as an indispensable tool for vaccine development and antiviral drug research. The initial coronavirus reverse genetics systems were generated based on a targeted RNA recombination strategy, which takes advantage of the high rate of homologous RNA recombination in CoVs [14–17]. However, this approach relies on viral replication and the requirement of virus passage mediated by viral replicase genes, which limits its ability to easily manipulate mutations across the entire genome. Over the past two decades, several creative methods have been established to successfully generate infectious  coronavirus clones. However, the enormous size of coronavirus genomes, combined with the instability and toxicity of certain viral sequences when cloned in bacterial hosts, represents a significant bottleneck for traditional reverse genetics approaches. Conventional systems relying on bacterial or yeast artificial chromosomes (BACs/YACs) [18, 19] or complex in vitro ligation strategies [20–22] are often laborious and prone to introducing unwanted deletions or mutations, impeding rapid progress [23, 24]. To circumvent these longstanding obstacles, cloning-free assembly methods have emerged as powerful alternatives. Among these, the circular polymerase extension reaction (CPER) has been reported as an attractive strategy, enabling the rapid, in vitro assembly of multiple DNA fragments into a single, infectious circular dsDNA for generating infectious viral clones [25–27]. The novel bacteria- or yeast-free method has been successfully used to establish infectious clones of SARS-CoV-2 [28, 29], enabling rapid and convenient manipulation of large viral genomes.

Reverse genetics systems that incorporate reporter genes have become indispensable tools in virology, enabling real-time visualization of viral dynamics and high-throughput screening for antiviral compounds. However, a robust and genetically stable reporter viral system for BCoV has remained a critical unresolved requirement, limiting progress in understanding its pathogenesis and vaccine development.

In this study, we established the first cloning-free reverse genetics system for BCoV via an optimized CPER method. We further validated its utility by generating a stable fluorescent reporter virus, which enables direct real-time visualization of viral replication and provides a useful tool for BCoV antiviral evaluation. Collectively, this work delivers a vital resource for advancing research on this critical veterinary pathogen.

## Materials and methods

### Cells, virus, antibodies, and compounds

BHK-21 and HRT-18G cells were maintained in Dulbecco’s modified Eagle medium (DMEM, Gibco) containing 10% fetal bovine serum (FBS, Gibco), 100 U/mL of penicillin, and 100 μg/mL of streptomycin. BHK-21 cells stably expressing the BCoV N gene were generated by lentiviral transduction followed by selection with G418 solution (400 μg/mL, Sigma). All the above cells were cultured at 37 °C under 5% CO_2_. The BCoV XJ2023 strain, isolated and preserved in our laboratory, was propagated in HRT-18G. Monoclonal antibodies against BCoV N and S proteins were generated in our laboratory by immunizing BALB/c mice with inactivated BCoV XJ2023. Compounds, including nafamostat (CAS: 82956-11-4), camostat (CAS: 59721-29-8), remdesivir (CAS: 1809249-37-3), molnupiravir (CAS: 2349386-89-4), nelfinavir (CAS: 159989-65-8), niclosamide (CAS: 50-65-7), nitazoxanide (CAS: 55981-09-4), ivermectin (CAS: 70288-86-7), ebselen (CAS: 60940-34-3), and baicalein (CAS: 491-67-8) were purchased from MCE and dissolved in 100% dimethyl sulfoxide (DMSO).

### Viral RNA extraction and cDNA synthesis

Total RNA was extracted from the BCoV-infected cells using TRIzol reagent (Vazyme) according to the manufacturer’s instructions. Thereafter, first-strand complementary DNA (cDNA) was synthesized using a HiScript II 1st Strand cDNA Synthesis Kit (Vazyme R212) with random hexamer primers and Oligo d(T). For viral RNA, the precise 5′ and 3′ termini of the viral genome were analyzed using the SMARTer RACE 5′/3′ Kit (Clontech) with specific primers (BCoV-5-Race and BCoV-3-Race, Additional file 1).

### Construction of BCoV fragment plasmids

Six overlapping cDNA fragments (F1–F6), up to 6000 base pairs in length and covering the entire BCoV genome, were amplified from synthesized cDNA using specific primers (Additional files 2 and 3) and PrimeSTAR GXL DNA polymerase (TaKaRa Bio). All six cDNA fragments were cloned into the pBR322 vector (pBR322-F1-F6). An untranslated region (UTR) linker containing the 3′ terminal 87 nucleotides (nt) of BCoV, BGH polyA, HDVr, CMV promoter, and the 5′ terminal 54 nt of BCoV was generated in pGEM-T. A silent mutation (T29409C) was introduced into pBR322-F6 as a genetic marker. For reporter virus construction, ZsGreen was inserted upstream of the ORF2a start codon in pBR322-F4.

### Optimizing CPER for production of coronavirus infectious double-stranded circular DNAs (ds-circDNAs)

BCoV recombinants were generated by CPER as described previously [29], with some modifications. Seven overlapping DNA fragments (F1–F6 and the UTR linker) were polymerase chain reaction (PCR)-amplified from corresponding plasmids using primers listed in Additional file 2 (Linker-Fw to -F6-Rv). Equimolar amounts of each fragment (0.02, 0.05, or 0.1 pM) were assembled in a 50 μL CPER reaction containing 2 μL PrimeSTAR GXL DNA polymerase. Cycling conditions were 98 °C for 5 min; 25 cycles of 98 °C for 10 s, 55 °C for 15 s, and 68 °C for 10 min; followed by 68 °C for 15 min. Reporter virus clones were assembled similarly using the F4-ZsGreen fragment.

### Rescue of recombinant BCoV

The 50 μL CPER reaction products were then used for transfection without further purification. Three independent rescue experiments (clones 1–3) were performed by transfecting BHK-21-N cells seeded in a six-well plate with Lipofectamine 3000 (Invitrogen), following the manufacturer’s protocol. At 24 h post-transfection, the cells were trypsinized and overlaid with a 70% confluent monolayer of HRT-18G cells, and the culture supernatants were replaced with DMEM containing 0.25 μg/mL trypsin (P0). Supernatants containing viruses were harvested when cytopathic effect (CPE) was evident (5–6 days post-transfection) and amplified once on HRT-18G cells to generate viral stocks with titers of ~1 × 10^7^ TCID_50_/mL determined by immunofluorescence assay (IFA) described below.

### Western blot

HRT-18G cells infected with CPER-derived BCoV, wtBCoV, or mock were lysed in NP40 buffer (Beyotime) containing phenylmethylsulfonyl fluoride (PMSF). Samples were separated by sodium dodecyl sulfate–polyacrylamide gel electrophoresis (SDS-PAGE), transferred to nitrocellulose membranes, and blocked with 5% bovine serum albumin (BSA)/TBST for 2 h at room temperature. Membranes were incubated overnight at 4 °C with anti-BCoV N or anti-GAPDH (Proteintech, 60004-1-Ig) antibodies diluted 1:1000, followed by horseradish peroxidase (HRP)-conjugated secondary antibodies (Proteintech, 1:5000). Signals were detected using enhanced chemiluminescence (ECL) reagent and imaged with an AI600 system.

### Indirect immunofluorescence assay

Infected cell monolayers were cultured in 12-well plates for 48 h and fixed with 4% formaldehyde for 20 min at room temperature, permeabilized with 0.1% Triton X-100 for 10 min, and blocked with 5% BSA/TBST for 1 h at room temperature. Cells were then incubated overnight at 4 °C with anti-BCoV N mAb 8E6 or naive mouse serum (1:1000), followed by fluorescein isothiocyanate (FITC)- or CoraLite594-conjugated goat anti-mouse IgG (Proteintech) for 1 h. Nuclei were stained with DAPI (1:1000), and images were captured using a Nikon inverted microscope or confocal microscope.

### Titration and growth kinetics

The infectious titers in the culture supernatants were determined by the 50% tissue culture infective dose (TCID_50_). The culture supernatants of cells were inoculated onto HRT-18G cells in 96-well plates after tenfold serial dilution with DMEM, and the infectious titers were determined at 72 h post-infection (hpi). For growth kinetics, BCoV was inoculated into HRT-18G cells in six-well plates at a multiplicity of infection (MOI) of 0.001 or 0.01, and the culture supernatants were replaced with new medium at 1 hpi and incubated for 72 h. The infectious titers in the culture supernatants of cells collected at 12, 24, 36, 48, 60, and 72 hpi were determined using the TCID_50_ assay.

### ZsGreen reporter gene stability assay

To assess reporter gene stability, rBCoV/Zs-T2A-ns2 was serially passaged in HRT-18G cells at an MOI of 0.01 for 15 passages. Cells and supernatants were harvested at 48 hpi, and 100 μL of diluted supernatant was used for each subsequent passage. Viral RNA was extracted every five passages to examine the integrity of the ZsGreen insertion by reverse transcription polymerase chain reaction (RT-PCR) using primers flanking the ns2 upstream region (forward: GTTGGTGACAGTTTGGTT, reverse: CTTCTTCAACTGAAGATTC). The expected product size was 871 base pairs (bp) for the wild-type sequence, and 1618 bp for the recombinant virus carrying the ZsGreen insert.

### Quantification of ZsGreen fluorescence intensity

To evaluate reporter virus replication kinetics and the antiviral compound screening assays, fluorescence intensity was quantitatively measured using an Infinite 200 PRO microplate reader (TECAN) equipped with i-Control software. HRT-18G cells infected with the reporter virus were cultured in Corning 96-well flat-bottom black plates to minimize background signal. Readings were performed in bottom-read mode with an excitation wavelength of 485 nm and an emission wavelength of 535 nm. To ensure accurate representation of the fluorescence across the entire well area and to maximize the signal-to-noise ratio, data were collected using 50 flashes per read with the “Multiple reads per well” function configured to a 2 × 2 circle-filled pattern.

### Cell viability assay

HRT-18G cells (1 × 10^4^) were seeded in 96-well plates for 20 h. A gradient dilution series of each compound was added to the cells, with five wells being used in parallel. After incubation for 48 h, the cells were incubated with CCK-8 reagent (Beyotime Biotechnology) for 45 min at 37 °C. The absorbance of each well at 450 nm was measured using the Infinite^®^ 200 PRO microplate reader. The cytotoxicity curves and CC_50_ values were generated using GraphPad Prism v8.0.1 software (GraphPad, La Jolla, CA, USA).

### Antiviral compound evaluation

HRT-18G cells were inoculated onto a 96-well plate (clear bottom) at a rate of 10,000 cells per well. After 24 h, the cells were washed twice with phosphate-buffered saline (PBS) (pH 7.2) and compounds were added at a final concentration of 5 μM per well. After 2 h, each well of cells was inoculated with 0.1 MOI of rBCoV/Zs-T2A-ns2. Subsequently, the cells were placed in a 37 °C incubator and cultured for 48 h. Finally, the cells were observed under an inverted fluorescence microscope (Nikon) and analyzed using the Infinite^®^ 200 PRO Microplate reader to measure fluorescent intensity.

### Time-of-addition assay

To investigate the stage of the BCoV life cycle targeted by the compounds, time-of-addition assays were performed using rBCoV/Zs-T2A-ns2, with DMSO-treated cells as controls. For the virucidal assay, compounds were incubated with virus at 37 °C for 2 h before inoculation onto HRT-18G cells, and viral titers were subsequently determined. For the adsorption assay, cells were pre-chilled at 4 °C for 1 h, then infected in the presence of compounds at 4 °C for 2 h to assess inhibition of viral attachment. For the entry assay, virus was first allowed to bind cells at 4 °C, unbound virus was removed, and pre-warmed compound-containing medium was added at 37 °C for 1–2 h; residual surface-bound virus was then removed with citrate buffer (pH 3.0), and intracellular viral RNA was quantified by reverse transcription quantitative polymerase chain reaction (RT-qPCR). For the replication assay, cells were infected for 2 h, and compounds were added at 4 hpi; viral RNA levels were measured at the indicated time points to evaluate the effects on post-entry replication.

### Statistical analysis

All the statistical analyses were performed via GraphPad Prism (GraphPad, version 8.0.1, USA). The data are presented as the means ± standard deviations (SDs) from at least three independent biological replicates. Multiple group comparisons were analyzed via one-way or two-way analysis of variance (ANOVA). Statistical significance was defined as follows: not significant (ns), *P* > 0.05, *P < 0.05, **P < 0.01, and ***P < 0.001.

## Results

### Cloning-free assembly of the full-length BCoV genome

**Figure 1:**
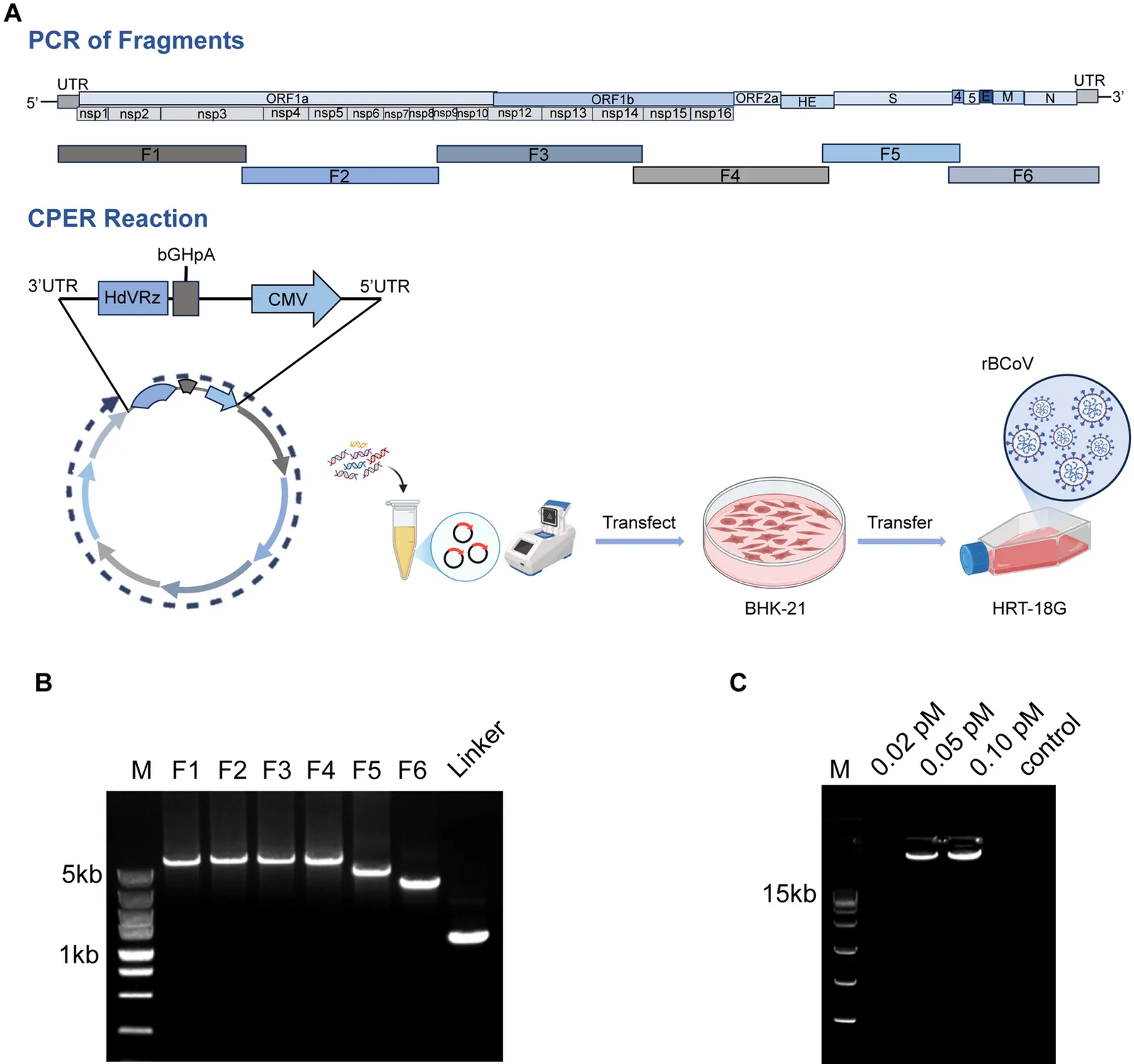
**Diagrams and workflow of the CPER technique for constructing infectious BCoV clones.** **A** Schematic diagram of the BCoV genome and CPER technique. The viral genome was divided into six overlapping fragments, each amplified with specific primers (Additional file 3). CMV: cytomegalovirus promoter; 5UTR: 5ʹ untranslated region; ORF1a, ORF1b, ORF2a, ORF4, ORF5: open reading frame 1a, 1b, 2a, 4, 5; HE: hemagglutinin esterase; S: spike; M: membrane; E: envelope; N: nucleoprotein; 3UTR: 3′ untranslated region; HDVr: ribozyme sequence of HDV; bGHpA: bovine growth hormone polyadenylation signal. **B** Seven PCR-amplified fragments generated from pBR322-F1–F6 and linker plasmids were analyzed by 1% agarose gel electrophoresis. **C** Agarose gel electrophoresis (0.8%) was performed to determine the optimal CPER conditions. Three concentrations of each cDNA fragment (0.02, 0.05, and 0.1 pM) were tested, and 0.1 pM was identified as the optimal concentration for generating the full-length circular BCoV cDNA. M, DNA size marker.

Given the large genome size (~31 kb) of bovine coronavirus (BCoV), we designed a circular polymerase extension reaction (CPER) strategy to assemble the full-length viral cDNA in vitro (Figure 1A). The BCoV genome was divided into six overlapping DNA fragments (F1–F6), each approximately 5–6 kb in size (Figure 1B). The F5 fragment contained the complete viral spike gene. A crucial component of our design is a 1135-bp linker fragment, which connects the 5′ and 3′ ends of the genome and incorporates essential elements for viral RNA transcription and replication. To promote viral RNA transcription in eukaryotic cells, the hepatitis delta virus ribozyme (HDVr) sequence was introduced to generate the authentic 3′ end of viral RNA, along with the cytomegalovirus (CMV) promoter for in vivo transcription of viral RNA by cell RNA polymerase II and the bGH poly(A) termination signal for efficient transcription termination. The linker sequence, from 5′ to 3′, is “BCoV-3′UTR-HDVr-bGH-CMV-BCoV 5′UTR”, and is denoted as BCoV-Linker (Figure 1A).

To assemble these seven fragments into a single circular DNA molecule, we optimized the CPER reaction conditions. Equimolar ratios of all seven fragments were mixed in a single tube with a high-fidelity DNA polymerase. The reaction was subjected to thermal cycling, allowing for sequential denaturation, annealing of overlapping ends, and polymerase extension, ultimately resulting in a seamless, double-stranded circular DNA. Furthermore, we determined that 0.1 pM for each fragment (F1 to F6, Linker) was the optimal DNA component concentration for the BCoV CPER reaction. Agarose gel electrophoresis of the CPER products revealed a distinct high-molecular-weight band migrating above the DNA ladder. This band putatively corresponded to the assembled full-length BCoV genome plus the linker, with definitive confirmation reliant on the subsequent successful rescue of the infectious virus (Figure 1C). These results demonstrate the successful establishment of a single-tube CPER method capable of efficiently assembling the entire BCoV genome into a transfection-ready circular DNA molecule, without the need for bacterial cloning.

### Efficient rescue of recombinant BCoV in BHK-21-N cell lines

To rescue infectious virus from the assembled genome, the CPER products without purification were directly transfected into either standard BHK-21 cells or a derivative cell line stably expressing the BCoV N protein (BHK-21-N) (Additional file 4). At 24 h post-transfection, the cells were digested with trypsin and co-cultured with an HRT-18G cell monolayer. Significant acceleration of virus recovery was observed in the BHK-21-N cells, where apparent viral infection was detected in the P1-generation cells. In contrast, rescue in standard BHK-21 cells required an additional passage (Additional file 5). This finding confirms that N protein co-expression significantly enhances the efficiency of coronavirus rescue. The rBCoV was subsequently propagated and titrated in HRT-18G cells, consistently yielding titers of ~10⁷ TCID₅₀/mL.

**Figure 2﻿:**
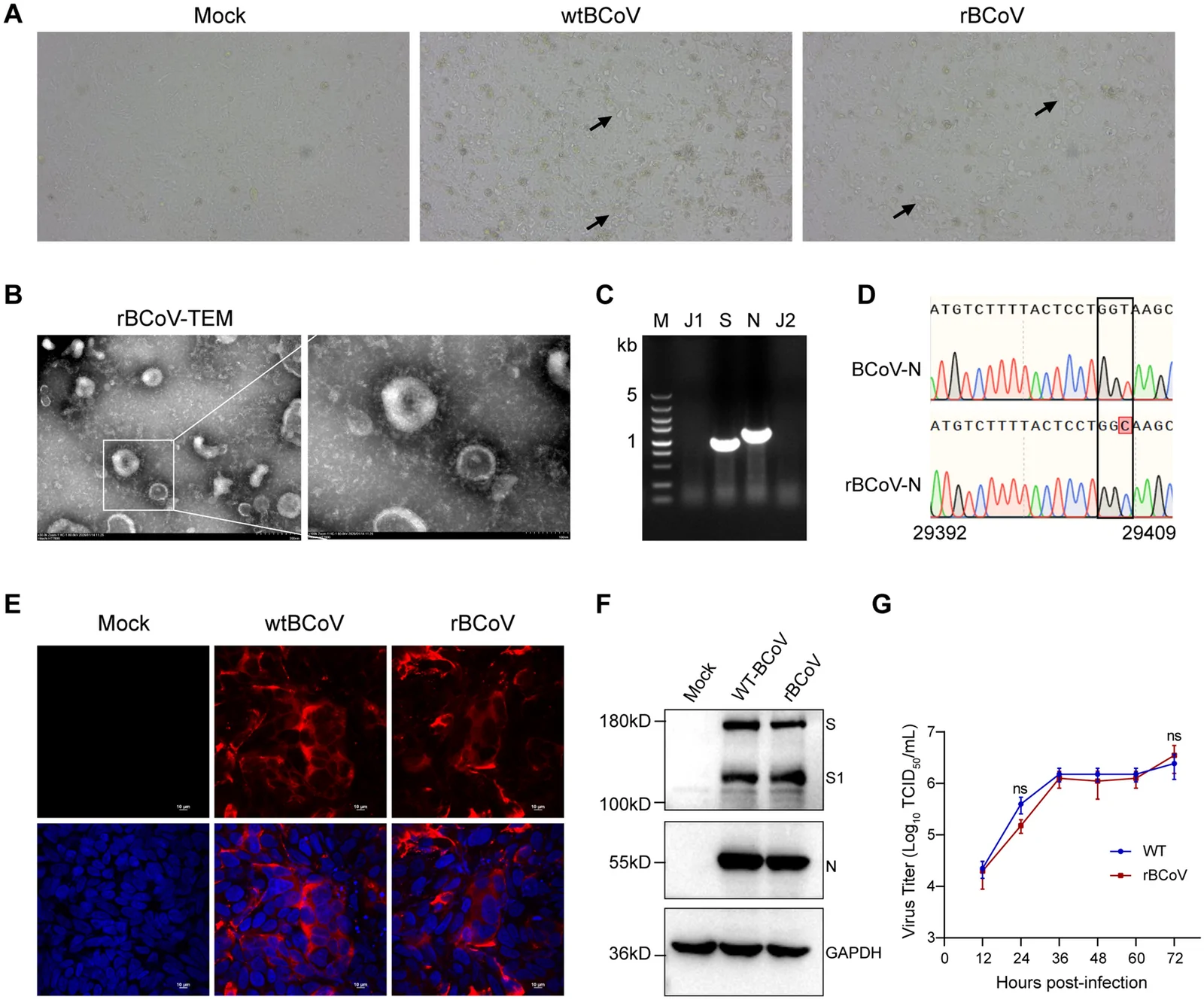
**Generation and in vitro characteristics of recombinant BCoV.** **A** Cytopathic effect (CPE) in HRT-18G cells with BCoV and rBCoV at 48 hpi. **B** Representative transmission electron microscopy (TEM) image of rBCoV particles in culture supernatant. Scale bar, 100 nm. **C**–**F** Successful rescue of recombinant BCoV was confirmed by RT-PCR (**C**), Sanger sequencing (**D**), indirect immunofluorescence assay (IFA) (**E**), and western blot (**F**). In particular, the viral protein (red) was detected using a specific N monoclonal antibody at 48 hpi, and the cell nuclei were stained with DAPI (blue) in IFA, then observed using a confocal microscope. Scale bar, 10 μm (**E**). **G** Multistep growth kinetics of the parental (WT) and recombinant (rBCoV) viruses in HRT-18G cells infected at an MOI of 0.01. Supernatants were collected at indicated time points for titration. The curves demonstrate comparable replication fitness. Data are presented as mean ± SD (*n* = 3); ns, not significant (two-way ANOVA). All panels in this figure present validation data from a single representative clone (rBCoV-clone-1). The reverse genetics rescue and subsequent phenotypic characterizations were independently performed three times with similar outcomes.

We conducted three independent CPER transfection experiments, all of which successfully yielded infectious viruses. This initial recovery demonstrates the high efficiency of the CPER platform for viral rescue, while simultaneously highlighting the robust replication capacity of the specific BCoV XJ2023 strain and the highly permissive nature of the HRT-18G host cells. For detailed phenotypic and genetic characterization, a representative clone (rBCoV-clone-1, hereafter referred to as rBCoV) was selected for subsequent assays. As shown in Figure 2, the cytopathic effect (CPE) induced by rBCoV—characterized by cell detachment and vacuole formation, gradually fusing to form syncytia—was identical to that of the wild-type BCoV (Figure 2A). Moreover, distinct viral particle morphology can be observed under the electron microscope (Figure 2B). RT-PCR with junction-spanning primers (Additional file 3) confirmed the precise joining of genomic fragments with the linker sequence, further validating the accuracy of the assembly (Figure 2C). To rigorously confirm the genomic integrity of the rescued BCoV and rule out unexpected large-scale deletions, we performed whole-genome sequencing (WGS). The Sanger sequencing and WGS analysis verified the full-length assembly, and all identified minor nucleotide variations as well as the synonymous mutation (T29409C) synthetically introduced into the viral nucleotide (N) gene have been detailed in Additional file 6 and Figure 2D.

### Confirmation of recombinant BCoV production

In order to confirm the recovery of rBCoV and compare its characteristics with those of wtBCoV, we performed a series of analyses of viral protein expression and replication kinetics. First, we assessed the viral protein expression in infected HRT-18G cells. IFA with an anti-N antibody confirmed N protein expression in cells infected with rBCoV and wtBCoV, but not in mock-infected cells. Crucially, DAPI nuclear staining unambiguously revealed the classic syncytial morphology: large, fused cytoplasmic areas (indicated by intense viral N protein fluorescence) containing multiple distinct, clustered nuclei (Figure 2E). Western blot was also performed to further validate these findings (Figure 2F). To compare viral fitness, HRT-18G cells were infected with rBCoV or wtBCoV at an MOI of 0.01, and supernatants were collected at the indicated time points for titration. Multistep growth curve analysis showed that rBCoV displayed replication kinetics comparable to those of the parental virus (Figure 2G). Collectively, these results confirm the successful rescue and replication of recombinant BCoV in vitro.

### Generation of a genetically stable reporter virus expressing ZsGreen

**Figure 3:**
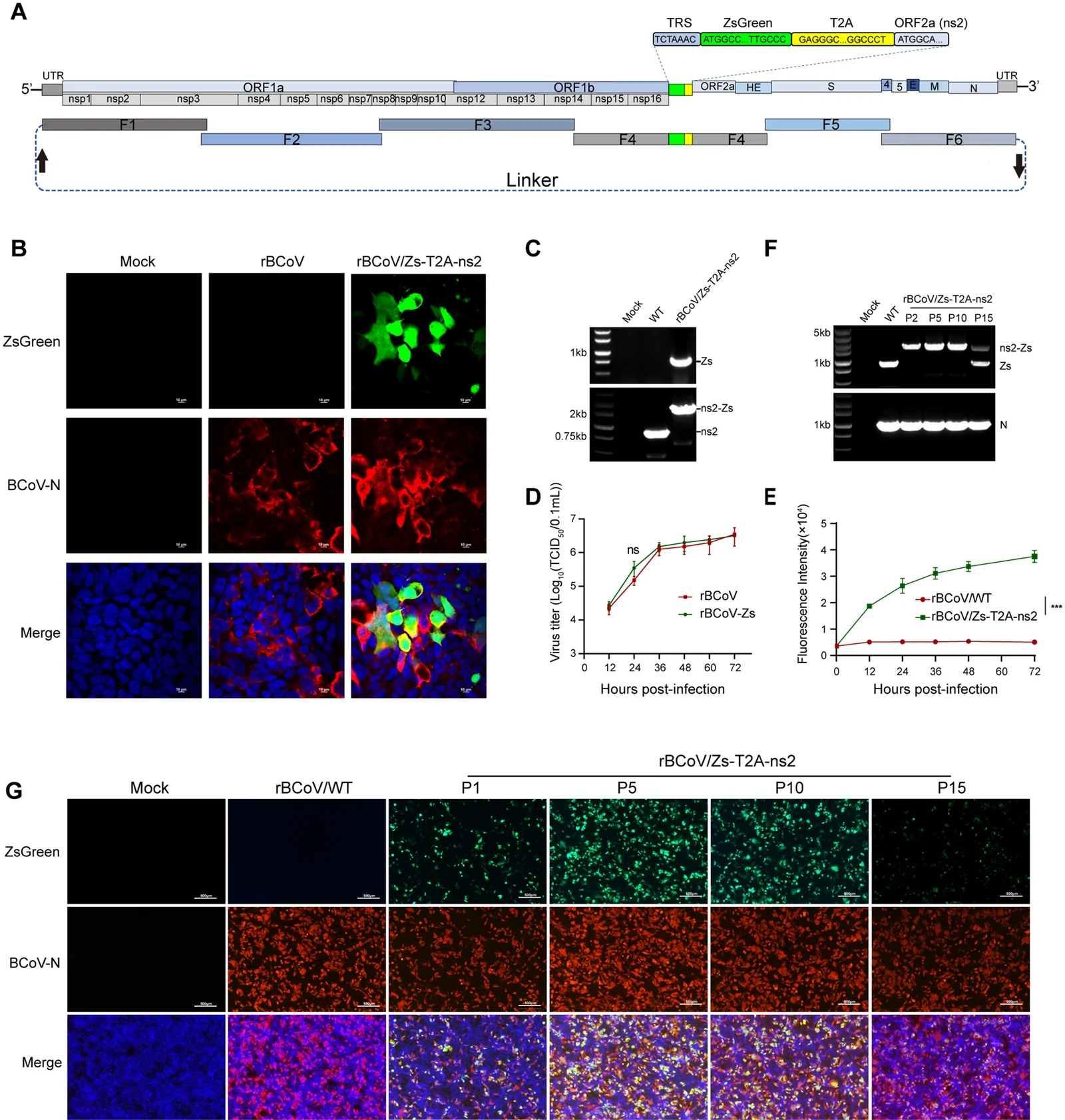
**Generation of rBCoV expressing ZsGreen fluorescent protein.** **A** Schematic of rBCoV/Zs-T2A-ns2 construction, with ZsGreen-T2A inserted upstream of ORF2a in the BCoV XJ2023 genome. **B** HRT-18G cells infected with WT or rBCoV/Zs-T2A-ns2 were analyzed by IFA for N protein expression at 48 hpi; nuclei were stained with DAPI. Scale bars, 10 μm. **C** RT-PCR detection of ZsGreen and the ORF1b–ns2 region in infected cells at 48 hpi. **D** Multistep growth curves of rBCoV and rBCoV/Zs-T2A-ns2. Supernatants were collected at indicated times for titration. Data are mean ± SD (*n* = 3); ns, not significant by two-way ANOVA with Dunnett’s test. **E** Fluorescence intensity of HRT-18G cells infected with rBCoV or rBCoV/Zs-T2A-ns2 was measured using a microplate reader (Infinite 200 PRO, TECAN). **F**, **G** Genetic stability of rBCoV/Zs-T2A-ns2 across serial passages was verified by RT-PCR and IFA (P1/P2, P5, P10, and P15). Scale bar, 500 μm.

Several engineered recombinant viruses expressing reporter genes have been developed for real-time monitoring of viral replication and high-throughput screening of viral inhibitors [30, 31]. Having confirmed the CPER system's efficacy for BCoV recovery, we engineered a recombinant BCoV expressing the ZsGreen fluorescent protein, a reporter known for its bright and stable signal. The ZsGreen gene was inserted upstream of the nonstructural protein 2 (ns2) gene, under the control of the authentic ns2 transcriptional regulatory sequence (TRS), separated by the self-cleaving T2A peptide derived from Thosea asigna virus. This strategy allows for bicistronic expression of both the endogenous protein (ns2) and ZsGreen from a single mRNA post-translationally, thereby linking fluorescence intensity directly to viral replication, making it an excellent tool for semiquantitative and visual monitoring of viral replication (Figure 3A). Fluorescence intensity was detected using an inverted fluorescence microscope (Nikon) and a microplate reader (Infinite 200 PRO, TECAN) to track the dynamics of viral replication in real time.

### BCoV ns2 is a stable site for exogenous gene expression

The reporter virus, designated as rBCoV/Zs-T2A-ns2, was successfully recovered using the above-described protocol. As shown in Figure 3B, HRT-18G cells infected with rBCoV/Zs-T2A-ns2 exhibited bright green fluorescence, which was absent in cells infected with the wild-type virus. Immunofluorescence co-staining further showed that ZsGreen-positive cells were also positive for the viral N protein, confirming that reporter expression is a reliable indicator of active viral infection. The genetic integrity of the insertion was verified by RT-PCR, which detected the expected larger amplicon spanning the ns2 region and the ZsGreen-T2A insertion specifically in rBCoV/Zs-T2A-ns2-infected cells (Figure 3C). Crucially, the insertion of the reporter cassette did not significantly impair viral fitness. A comparative multistep growth curve analysis showed that the replication kinetics of rBCoV/Zs-T2A-ns2 were nearly identical to the rBCoV, with no statistically significant differences observed at any time point post-infection (Figure 3D). Furthermore, we conducted a quantitative analysis of the fluorescence signal over time to validate the utility of this reporter gene. Consistent with the viral growth curve, we observed a time-dependent increase in ZsGreen fluorescence intensity, which accurately reflected the progression of viral replication (Figure 3E). Next, we examined the genetic stability of rBCoV/Zs-T2A-ns2 during serial passages in HRT-18G cells. Complete and stable ZsGreen expression was maintained through P1 to P10, as no deletions were detected at the insertion site, indicating high genetic stability. However, a significant reduction in fluorescence was observed at P15, suggesting the eventual loss of the foreign gene under prolonged replication pressure (Figure 3F, G). To conclude, the rBCoV/Zs-T2A-ns2 virus is well suited for visualizing viral replication kinetics, and the ns2 locus of BCoV is a potential site to accommodate exogenous genes.

### Antiviral compound evaluation using rBCoV/ Zs-T2A-ns2

Currently, there are no clinically approved specific treatments for BCoV infection. To validate the practicality of our reporter system in antiviral assays, we evaluated the inhibitory effects on BCoV replication of a panel of ten US Food and Drug Administration (FDA)-approved antiviral compounds with diverse mechanisms of action. The selected compounds comprised viral entry inhibitors (nafamostat, camostat) [32, 33], viral polymerase inhibitors (remdesivir, molnupiravir) [34], a coronavirus 3CL protease inhibitor (nelfinavir) [35], and several host-directed antiviral agents (niclosamide, nitazoxanide, ivermectin, ebselen, and baicalein) [36–40]. Remdesivir, which has been widely reported to exhibit broad-spectrum activity against coronaviruses [41–43], was used as a positive reference control.

**Figure 4:**
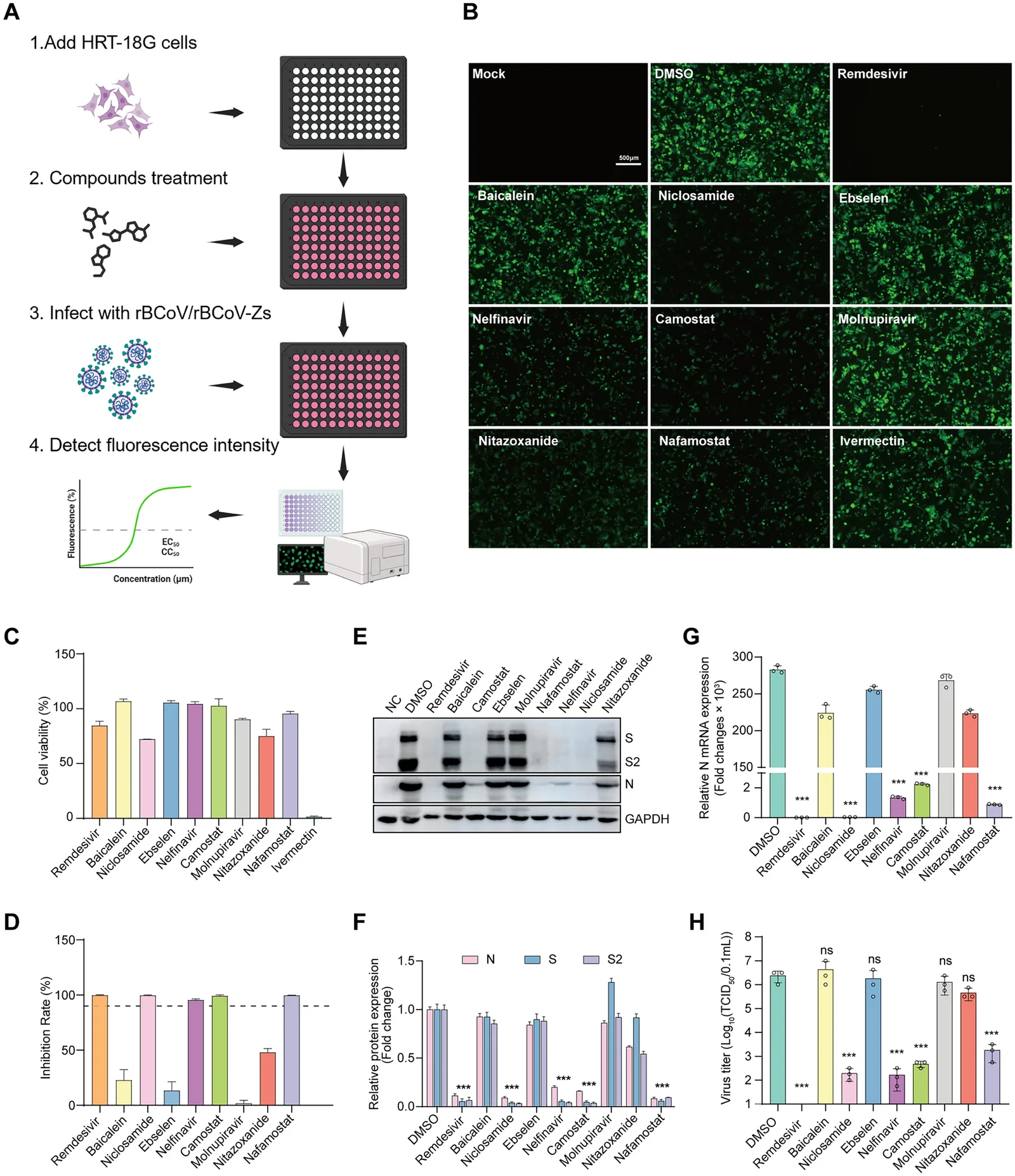
**Identification of antiviral compounds against rBCoV/Zs-T2A-ns2.** **A** Schematic of the antiviral screening workflow. HRT-18G cells were pretreated with compounds (5 μM) for 2 h and infected with rBCoV/Zs-T2A-ns2 (0.1 MOI). Fluorescence intensity was measured at 48 hpi, and inhibition rates were calculated relative to DMSO-treated infected and mock-infected controls. **B** Primary screening of ten compounds against rBCoV/Zs-T2A-ns2 infection based on fluorescence inhibition at 48 hpi. **C** Cytotoxicity of ten candidate compounds in HRT-18G cells was assessed by CCK-8 assay after 48 h of treatment with the compounds. Data were presented as a percentage relative to the DMSO-treated control group. **D** BCoV inhibition rate of ten candidate compounds in HRT-18G cells. Data were presented as a percentage relative to the DMSO-treated control group. **E** Western blot analysis of viral S, S2, and N proteins in infected cells treated with nine selected compounds; GAPDH served as a loading control. **F** Quantification of viral protein expression in **E** using ImageJ software. **G** Relative viral N mRNA levels were determined by RT-qPCR at 48 hpi. **H** Viral titers in culture supernatants were measured by TCID_50_ assay. Data are mean ± SD (*n* = 3). Statistical significance was analyzed by one-way ANOVA with Dunnett’s test versus DMSO control; ns, not significant; **P < 0.01, ***P < 0.001.

A primary screen was conducted at a final concentration (5 µM) to rapidly identify potent viral inhibitors. Following a preliminary cytotoxicity assessment which excluded ivermectin due to its significant toxicity in HRT-18G cells (Figure 4C and Additional file 7), four compounds—camostat, nafamostat, niclosamide, and nelfinavir—were identified alongside the positive control remdesivir as preliminary hits, each showing significant antiviral activity with 50% inhibition of ZsGreen expression (Figure 4B). While fluorescence intensity measurements revealed that these compounds strongly suppressed viral-driven ZsGreen expression (Figure 4D), the antiviral potential of specific hits requires cautious interpretation. Notably, the 25–30% reduction in cell viability observed with niclosamide could induce cellular stress responses that indirectly contribute to the observed antiviral effect. Nevertheless, the reduction in fluorescence observed in the reporter assay directly correlated with a corresponding decrease in viral N protein expression (Figure 4E, F) and N gene mRNA levels (Figure 4G) in infected cells, as well as viral titers (Figure 4H). Ultimately, while the translational viability of certain individual hits may be limited, these results definitively confirm that the ZsGreen signal is a highly reliable indicator of viral replication.

**Figure 5﻿:**
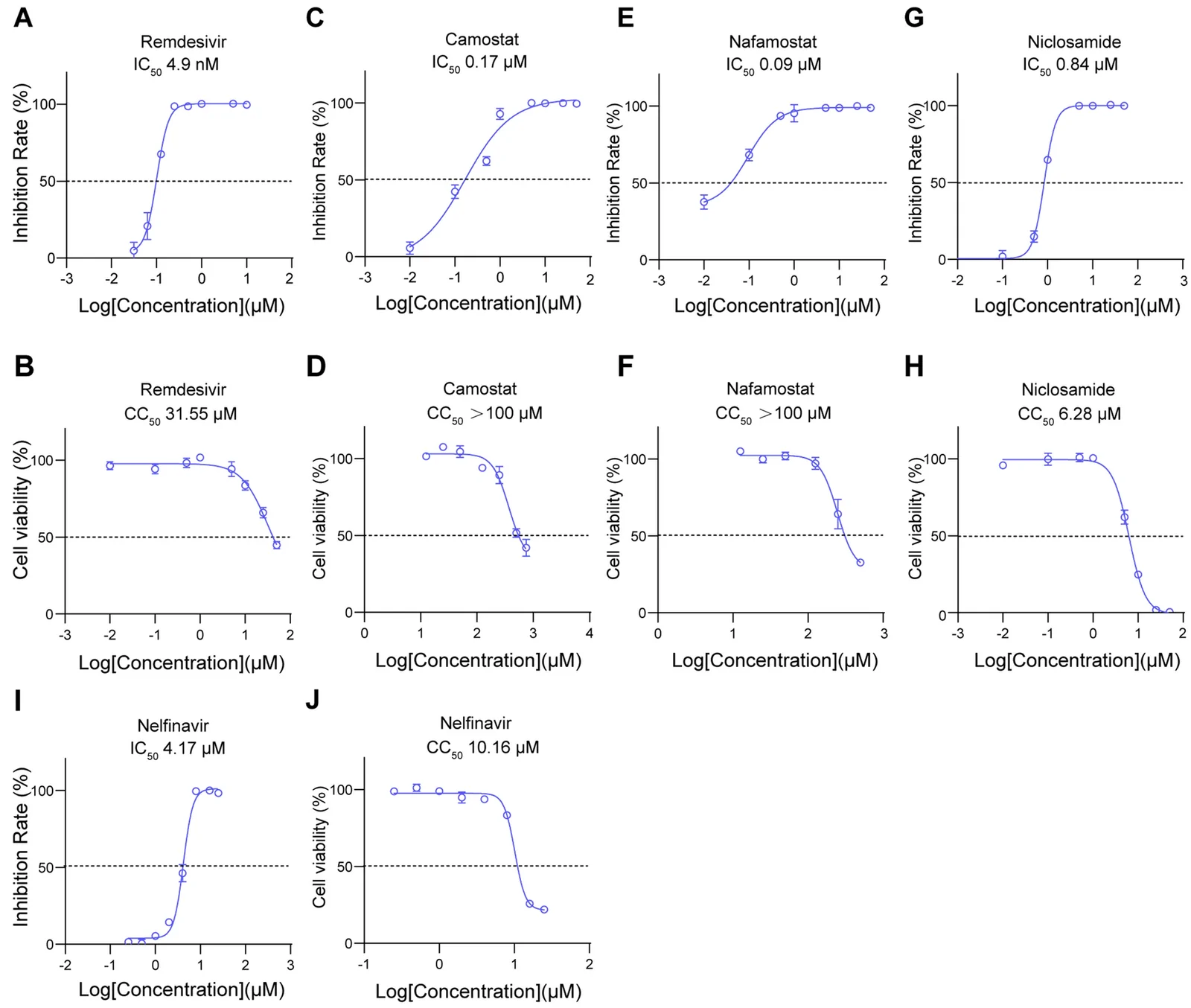
**IC**_**50**_** and CC**_**50**_** of five screened compounds against BCoV infection.** Dose–response curves for antiviral activity and cytotoxicity of remdesivir, camostat, nafamostat, niclosamide, and nelfinavir were determined in HRT-18G cells infected with rBCoV/Zs-T2A-ns2. Antiviral efficacy (**A**, **C**, **E**, **G**, **I**) was assessed by fluorescence intensity at 48 hpi and used to calculate IC_50_ values. Cytotoxicity (**B**, **D**, **F**, **H**, **J**) was measured by CCK-8 assay to determine CC_50_ values. Data are mean ± SD (*n* = 3).

A detailed dose-dependent antiviral analysis was conducted to further evaluate the antiviral activity of these five candidate compounds. The fluorescence activity inhibition rates of these compounds were evaluated, revealing that the five compounds exhibited 50% inhibitory concentrations (IC_50_) below 5 μM (Table 1). Additionally, based on the CCK-8 cell viability testing results, the 50% cytotoxic concentration (CC_50_) of these five compounds was determined to be greater than 5 μM (Figure 5A–J), indicating that their antiviral effects were barely affected by cytotoxicity. These results suggest that remdesivir, camostat, nafamostat, nelfinavir, and niclosamide have potential as antiviral agents for controlling BCoV infections in vitro.

**Table 1:** **CC**_**50**_**, IC**_**50**_**, and SI of the five compounds**

Hit compounds	CC_50_ (μM)	IC_50_ (μM)	Selectivity index (SI)
Remdesivir	31.55	0.0049	6438.78
Camostat	> 100	0.17	> 588.24
Nafamostat	> 100	0.09	> 1111.11
Niclosamide	6.28	0.84	7.48
Nelfinavir	10.16	4.17	2.44

### Effects of the identified hit compounds on different stages of BCoV infection

**Figure 6﻿:**
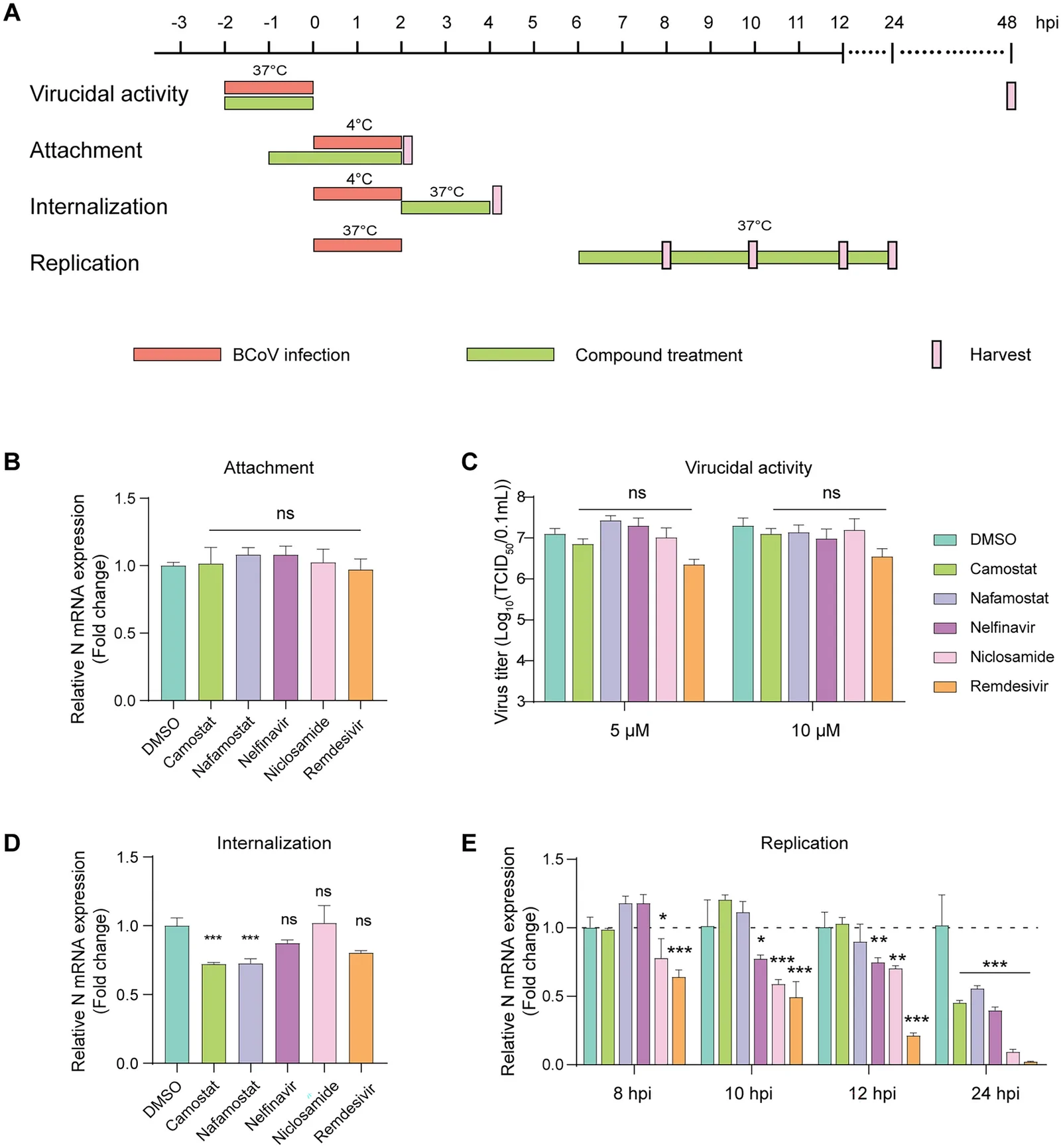
**Time-of-addition assay of five hit compounds against BCoV infection in HRT-18G cells.** **A** Schematic of the time-of-addition experiment. Orange-red bars represent BCoV infection; green bars represent camostat, nafamostat, nelfinavir, niclosamide, and remdesivir treatment; vertical bars represent the time points of cell harvesting. **B** HRT-18G cells were infected with rBCoV/Zs-T2A-ns2 (MOI = 1) and treated with compounds (5 μM) during the stage of viral attachment. The relative N mRNA expression was subsequently analyzed by RT-qPCR. **C** For virucidal activity, rBCoV/Zs-T2A-ns2 (0.1 MOI) was incubated with each compound (5 μM and 10 μM) at 37 °C for 2 h, and the mixture was then added to the cells. The supernatant was harvested to determine the viral titers at 48 hpi. **D** HRT-18G cells were infected with rBCoV/Zs-T2A-ns2 (MOI = 1) and treated with each compound (5 μM) during the internalization stage. **E** HRT-18G cells infected with rBCoV/Zs-T2A-ns2 (MOI = 0.1) were treated with each compound (5 μM) at 4 hpi, and viral N mRNA was measured 2, 4, 6, and 18 h later. Data are mean ± SD (*n* = 3). *P* < 0.05 (*), *P* < 0.01 (**) and *P* < 0.001 (***).

To determine which stages of the BCoV infection cycle were affected by camostat, nafamostat, nelfinavir, niclosamide, and remdesivir, we performed a time-of-drug-addition assay to assess their effects on virucidal activity, attachment, internalization, and replication (Figure 6A). As shown in Figure 6B, C, and Additional file 8A, at concentrations of both 5 µM and 10 µM, none of the tested compounds exhibited direct virucidal activity or influenced the attachment of BCoV to host cells. However, during the internalization phase (Figure 6D and Additional file 8B), nelfinavir and niclosamide had no effect, whereas camostat and nafamostat significantly inhibited viral internalization. At the early stages of replication, nelfinavir, niclosamide, and remdesivir markedly suppressed BCoV replication, while camostat and nafamostat showed no inhibitory effect (Figure 6E). Taken together, these findings suggest that remdesivir, nelfinavir, and niclosamide interfere with BCoV replication, whereas camostat and nafamostat primarily target the internalization step of the viral life cycle.

## Discussion

BCoV is an animal pathogen with substantial economic importance. It frequently contributes to complex co-infections with other enteric pathogens, such as rotavirus, in clinical settings [44–46], which complicates in vitro isolation and propagation. In this study, we report the development of the first cloning-free reverse genetics system for the BCoV XJ2023 strain and successfully rescued recombinant BCoV expressing ZsGreen fluorescent proteins based on CPER. Furthermore, we demonstrated the remarkable genetic stability of the reporter viruses and validated their utility for antiviral compound evaluation.

A key aspect of coronavirus reverse genetics is genomic stability. As a member of the RNA viruses, the coronavirus is prone to gene mutations or deletions during in vitro recombination [47]. Compared with conventional reverse genetics platforms, such as those relying on BAC/YAC systems or in vitro ligation/transcription, our optimized CPER-based strategy offers marked advantages in genetic integrity and efficiency. In our study, we designed a BCoV-specific linker fragment, which seamlessly joins the viral 5′ and 3′ ends via a simple PCR procedure while incorporating all necessary transcriptional elements. This design completely bypasses the need for bacterial cloning or in vitro transcription, thus eliminating laborious screening steps and minimizing the risk of introducing unwanted mutations. Our successful recovery of rBCoV/Zs-T2A-ns2 further demonstrates the broad applicability of the specific linker for bovine coronavirus rescue. Moreover, our use of BHK-21-N cells, which were derived from BHK-21 cells and previously shown to increase viral RNA replication/transcription, contributed to the increased yield of viral particles (Additional file 5) [28]. It is worth noting that, although some reports indicate that BCoV cell-adapted strains can replicate in Vero and MDBK (Madin–Darby bovine kidney) cells [48], the XJ2023 strain exhibits remarkably low replication efficiency in these cell lines, with only sporadic infections observed in MDBK cells and virtually no replication in Vero cells (Additional file 9).

Phenotypic analysis revealed that the unmodified recombinant virus exhibited similar growth kinetics to the wild-type virus. Generally, the insertion of exogenous genes at certain sites of coronavirus leads to a significant attenuation of viral replication or virulence, a phenomenon that has been previously reported and employed in the generation of attenuated viruses [49, 50]. For monitoring viral replication, we intended to identify an exogenous gene insertion site that permits stable foreign gene expression without compromising viral fitness. Specifically, rBCoV/Zs-T2A-ns2 was successfully rescued by inserting the reporter gene upstream of the ns2 gene. The ns2 protein is a 30 kDa nonstructural protein and one of the accessory proteins of BCoV, expressed from subgenomic mRNA in the cytoplasm of infected cells. Homologous proteins are also encoded in other closely related group 2a coronaviruses, and these proteins collectively constitute a subset of a large superfamily of 2H phosphodiesterase proteins [11]. We selected the ns2 gene, as its phosphodiesterase (PDE) activity is crucial for the virus to antagonize type I interferon and cause hepatitis [12, 13], but it is not essential for viral replication and packaging in vitro [51], making it a pathogenically relevant site for modification. Furthermore, the T2A peptide strategy ensures co-expression of the reporter protein and ns2 while maintaining their functional separation, thereby minimizing interference with the normal viral life cycle. Consequently, serial passaging for more than ten passages demonstrated that the reporter virus exhibited excellent genetic stability, with no loss or attenuation of reporter gene expression, confirming the robustness of the insertion site and establishing a reliable tool for visualizing and quantifying viral infection dynamics. Future studies in appropriate animal models will be important to further validate the feasibility of this platform in vivo and to assess its utility for investigating BCoV pathogenesis.

The incorporation of easily detectable reporter genes into viral genomes has greatly advanced studies of viral replication, virus–host interactions, antiviral and neutralizing antibody evaluation, and in vivo imaging. Reporter systems with diverse designs, including dual-reporter [52], conditionally replicating [53], and pseudovirus platforms [54], have become indispensable tools in modern virology research. Well-established reporter systems have been constructed for a range of viruses, including influenza virus, coronavirus, HIV, and hepatitis C virus (HCV) [31, 55]. Recently, Sugiura et al. reported a BAC-derived BCoV reporter virus by replacing the ORF2 (ns2) gene with ZsGreen, which provided valuable biological insights but resulted in attenuated peak viral titers [56]. In our study, to generate a reporter virus suitable for robust antiviral evaluation without compromising viral fitness, we utilized a T2A-mediated co-expression strategy. By inserting ZsGreen upstream of the ns2 gene rather than replacing it, our rBCoV/Zs-T2A-ns2 maintains robust replication kinetics comparable to those of the wild-type virus and demonstrates high genetic stability, enabling highly sensitive and quantitative monitoring of viral replication with strong signal and low background. Compared with conventional CPE- or titration-based methods, this system allows more rapid assessment of viral replication and can be readily adapted for larger-scale antiviral evaluation. Using this platform, we identified several FDA-approved antiviral compounds, including camostat, nafamostat, nelfinavir, and niclosamide, which dose-dependently inhibited BCoV replication. However, their immediate potential as clinical antivirals requires cautious interpretation. For instance, although niclosamide exhibited antiviral activity, it also induced a 25–30% reduction in cell viability at effective concentrations. This moderate cytotoxicity could induce cellular stress responses that indirectly contribute to the observed viral inhibition. Similarly, the very narrow selectivity index (SI ≈ 2) of nelfinavir limits its translational potential against this specific virus. These results demonstrate that our reporter system enables real-time, quantitative, and effective evaluation of antiviral efficacy, providing a powerful tool for therapeutic development.

The compounds identified in this study all possess unique antiviral mechanisms. Our time-of-addition experiments indicate that nelfinavir and niclosamide primarily act at the replication stage of BCoV infection, whereas camostat and nafamostat interfere with viral internalization. Nelfinavir has been found to bind reversibly to the active site of the coronavirus 3CL protease, competitively inhibiting its enzymatic activity and thereby blocking viral replication [57]. Niclosamide acts as a proton carrier and targets acidic endosomes, exhibiting broad-spectrum antiviral activity [58]. Furthermore, it interferes with host metabolic and signaling pathways, contributing to the suppression of coronaviruses [59]. These host-directed mechanisms align with our findings that nelfinavir and niclosamide exert replication-stage inhibition against BCoV. By contrast, camostat has been reported to specifically inhibit the host transmembrane serine protease TMPRSS2, thus blocking the cleavage and activation of the coronavirus spike protein and preventing the virus from entering host cells [33]. Similarly, in addition to covalently inhibiting TMPRSS2, nafamostat is also capable of inhibiting the furin protease, thereby reducing the pre-activation of the spike protein, and exhibits more potent and broad-spectrum antiviral activity. Therefore, we speculate that the internalization inhibition we observed may be related to the disruption of viral membrane fusion. Additionally, remdesivir exerts its potent antiviral activity exclusively during the post-entry replication stage, which perfectly aligns with its established mechanism as a nucleoside analogue targeting the viral RdRp [20, 60].

Interestingly, while molnupiravir has demonstrated potent broad-spectrum efficacy against various coronaviruses, including SARS-CoV-2 [61], our initial screening at 5 µM showed an apparent lack of activity against BCoV XJ2023. Subsequent dose–response evaluations (from 5 µM to 200 µM) revealed that complete inhibition of BCoV replication by molnupiravir requires a remarkably high concentration (greater than 50 µM) (Additional file 10). This reduced sensitivity suggests that BCoV may possess distinct viral replication dynamics or highly efficient proofreading exonuclease (ExoN) activity that mitigates the incorporation of mutagenic nucleoside analogues at lower concentrations. This highlights the need for alternative, highly potent antiviral candidates for effective BCoV intervention.

These complementary, stage-specific antiviral mechanisms described above support potential combination strategies and offer mechanistic insights for future antiviral optimization in the livestock farming industry. In future studies, this platform could be expanded for higher-throughput antiviral screening, enabling the systematic evaluation of larger compound libraries. This may help identify additional and potentially more potent inhibitors of BCoV replication.

In summary, we have successfully established an efficient, flexible, and robust CPER-based reverse genetics system for BCoV. This platform substantially advances BCoV research by overcoming previous technical barriers to in vitro viral manipulation, enabling the rapid generation of recombinant and reporter viruses. By providing an indispensable tool for investigating gene function, screening antiviral agents, and potentially developing new-generation vaccines, this work lays a solid foundation for advancing our understanding of BCoV pathogenesis and accelerating control of this economically important disease.

## Supplementary Information

**Additional file 1.** **Primers used in this article for RACE.**

**Additional file 2.** **Information on BCoV XJ2023 strain cDNA fragments for CPER.**

**Additional file 3.** **Primers used for BCoV reconstruction and identification.**

**Additional file 4.** **Generation and characterization of BHK-21 cells stably expressing the BCoV N protein.** (A) N mRNA levels in control BHK-21 cells and ten monoclonal cell lines were measured by RT-qPCR and normalized to GAPDH. Data are mean ± SD (*n* = 3). One-way ANOVA with Dunnett’s test: ns, not significant; *P < 0.05, **P < 0.01, ***P < 0.001. (B) N protein expression in control cells and clones #7 and #10 was analyzed by western blot. (C) N protein expression was visualized by IFA. Scale bars, 500 μm.

**Additional file 5.** **Stable expression of the BCoV N protein enhances the efficiency of recombinant virus recovery.** (A) Representative fluorescence images showing the recovery of rBCoV/Zs-T2A-ns2 in parental BHK-21 cells and BHK-21-N cells at the initial recovery stage (P0) and after the first (P1) and second (P2) blind passages. Viral recovery was monitored by ZsGreen fluorescence. Scale bar, 500 μm. (B) Infectious virus titers recovered from parental BHK-21 and BHK-21-N cells at P0, P1, and P2 were determined by TCID_50_ assay. Data are presented as mean ± SD (*n* = 3).

**Additional file 6.** **Differences between BCoV XJ2023-WT and rBCoV.**

**Additional file 7.** **Ivermectin exhibits significant toxicity to HRT-18G.** HRT-18G cells were treated with ivermectin for 48 h, and cytotoxicity was measured by CCK-8 assay. Data are mean ± SD (*n* = 3).

**Additional file 8.** **Time-of-addition assay evaluating hit compounds at 10 µM.** HRT-18G cells were infected with rBCoV/Zs-T2A-ns2 (MOI = 1) and treated with compounds (10 μM) during the stage of viral attachment (A) or the internalization stage (B). The relative N mRNA expression was subsequently analyzed by RT-qPCR. Data are mean ± SD (*n* = 3). *P* < 0.05 (*), *P* < 0.01 (**) and *P* < 0.001 (***).

**Additional file 9.** **Replication of BCoV XJ2023 in different cell lines.** (A) Relative viral N mRNA levels in HRT-18G, Vero, and MDBK cells infected with BCoV XJ2023 (MOI = 0.1) at 48 hpi were determined by RT-qPCR and normalized to GAPDH. Data are presented as mean ± SD (*n* = 3). Statistical significance was analyzed by one-way ANOVA with Dunnett's multiple comparisons test. ns, not significant; ***P < 0.001. (B) Viral N protein expression in HRT-18G, Vero, and MDBK cells at 48 hpi was analyzed by western blot using a monoclonal antibody against the BCoV N protein. (C) Representative fluorescence images of HRT-18G, Vero, and MDBK cells infected with rBCoV/Zs-T2A-ns2 at 48 hpi. Viral infection was visualized by ZsGreen fluorescence. Scale bar, 500 μm.

**Additional file 10.** **Molnupiravir requires a higher concentration to inhibit BCoV.** A broad concentration gradient (5, 10, 20, 40, 50, 80, 100, and 200 µM) of molnupiravir was tested against BCoV XJ2023, and antiviral efficacy was assessed by monitoring ZsGreen fluorescence (A), RT-qPCR (B), and western blot (C).

## Data Availability

All data generated during this study are included in this published article and its additional files.
