# Supplementary material for: An efficient and versatile reverse genetics system for bovine coronavirus facilitates antiviral evaluation
Source: Vet Res. 2026 Oct 11;57:180. doi: 10.1186/s13567-026-01851-6 (PMC13654213; doi:10.1186/s13567-026-01851-6)
Supplement: Supplementary file 1 [file 13567_2026_1851_MOESM1_ESM.docx]

**Additional file 1. Primers used in this article for RACE.**

| **Name** | **Oligo sequences (5ʹ-3ʹ)** | **Application** |
| --- | --- | --- |
| BCoV-5-Race | GATTACGCCAAGCTTAACTGCATAACAAAACCACAATCTTTCAAATCAACACCAT | RACE |
| BCoV-3-Race | GATTACGCCAAGCTTACACCTGATATGGCTGATCAAATTGCTAGTCTTGTTCTGG |  |
