# Supplementary material for: An efficient and versatile reverse genetics system for bovine coronavirus facilitates antiviral evaluation
Source: Vet Res. 2026 Oct 11;57:180. doi: 10.1186/s13567-026-01851-6 (PMC13654213; doi:10.1186/s13567-026-01851-6)
Supplement: Supplementary file 2 [file 13567_2026_1851_MOESM2_ESM.docx]

**Additional file 2. Information of BCoV XJ2023 strain cDNA fragments for CPER.**

| **Fragment** | **Start** | **End** | **Oligos** | | **Template** | **Size** |
| --- | --- | --- | --- | --- | --- | --- |
| Linker | 1 | 1135 | Linker-F | Linker-R | Linker plasmid | 1135 bp |
| BCoV-F1 | 1101 | 7029 | F1-Fw | F1-Rv | pBR322-F1 Plasmid | 5929 bp |
| BCoV-F2 | 6990 | 13094 | F2-Fw | F2-Rv | pBR322-F2 Plasmid | 6105 bp |
| BCoV-F3 | 13055 | 19015 | F3-Fw | F3-Rv | pBR322-F3 Plasmid | 5961 bp |
| BCoV-F4 | 18976 | 24718 | F4-Fw | F4-Rv | pBR322-F4 Plasmid | 5743 bp |
| BCoV-F4-Zs | 18976 | 25465 | F4-Fw | F4-Rv | pBR322-F4-Zs Plasmid | 6490 bp |
| BCoV-F5 (Spike) | 24679 | 28821 | F5-Fw | F5-Rv | pBR322-F5 Plasmid | 4143 bp |
| BCoV-F6 | 28784 | 35 | F6-Fw | F6-Rv | pBR322-F6 Plasmid | 3229 bp |
