# Supplementary material for: An efficient and versatile reverse genetics system for bovine coronavirus facilitates antiviral evaluation
Source: Vet Res. 2026 Oct 11;57:180. doi: 10.1186/s13567-026-01851-6 (PMC13654213; doi:10.1186/s13567-026-01851-6)
Supplement: Supplementary file 3 [file 13567_2026_1851_MOESM3_ESM.docx]

**Additional file 3. Primers used for BCoV reconstruction and identification.**

| **Name** | **Oligo sequences (5ʹ-3ʹ)** | | **Application** |
| --- | --- | --- | --- |
| Linker-F | ATTATGGCCAATTGGAAGAATCACaaaaaaaaaaaaaaaaaaaaaaaaaaaaaaGGGTC | | Linker fragment construction |
| Linker-R | GAAGCGGGATGCACGCACGCAAATCGCTCACAATCACGGTTCACTAAACGAGCTCTGCT | |  |
| F1-Fw | GATTGTGAGCGATTTGCGTGCGTGCATCCCGCTTCACTGA | | F1 construction |
| F1-Rv | TAATAATACGCTGAGTATAATACTTACCTCCGTCACAATA | |  |
| F2-Fw | TATTGTGACGGAGGTAAGTATTATACTCAGCGTATTATTA | | F2 construction |
| F2-Rv | CTGTAAAGCCTGCAAAACAGTATTGTCCTTTGCGTAATCG | |  |
| F3-Fw | CGATTACGCAAAGGACAATACTGTTTTGCAGGCTTTACAG | | F3 construction |
| F3-Rv | ACTACATTGAACTCTCGTTTCAACGGCCTGTGGCACTTTA | |  |
| F4-Fw | TAAAGTGCCACAGGCCGTTGAAACGAGAGTTCAATGTAGT | | F4 construction |
| F4-Rv | CATGCAGCCTAGTACCATTATCCACCATAAAATATAACAA | |  |
| F5-Fw | TTGTTATATTTTATGGTGGATAATGGTACTAGGCTGCAT | F5 construction | |
| F5-Rv | AGCAGGCATTGTGGTAGCTATTATAATATGCTCGACCT |  |  |
| F6-Fw | AGGTCGAGCATATTATAATAGCTACCACAATGCCTGCT | F6 construction | |
| F6-Rv | TTTTTTTTTTTGTGATTCTTCCAATTGGCCATAATTAACTTCATT |  |  |
| ORF1ab-Rv | CCGTGCTTGGACTGGGCCATTCTTAAAGTTTAGATTACATTAACC | F4-Zs fusion | |

**Continued Table S3. Primers used for BCoV reconstruction and identification**

| **Name** | **Oligo sequences (5ʹ-3ʹ)** | | **Application** |
| --- | --- | --- | --- |
| ZsGreen-Fw | ATGTAATCTAAACTTTAAGAATGGCCCAGTCCAAGCACGGCCTGA | F4-Zs fusion | |
| T2A-Zs-R | TCTGCATAAGCGACTGCCATAGGGCCGGGATTCTCCTCCACGTCA |  |  |
| ORF2a-Fw | TGGAGGAGAATCCCGGCCCTATGGCAGTCGCTTATGCAGACAAGC |  |  |
| J1-Fw | AAAAGCACCACACCCCACGCAAAAACAAGT | Connection detection | |
| J1-Rv | GACATACCTAGAGGGGGTGTAACCAAAACT |  |  |
| BCoV-S-Fw | TTGTTTGTGCACCCCAGACCCCATTACATC | rBCoV S gene detection | |
| BCoV-S-Rv | ACTTGCAACTGTGTAGTGTCAAGTAATTCA |  |  |
| BCoV-N- Fw | GTTTACACCTGGACTGCTGATGAAGCTATT | rBCoV N gene detection | |
| BCoV-N-Rv | AGGCTCTACTGGATGCGCGTGAAGTAGATC |  |  |
| J2-Fw | CTTGGAACTAGTGATCCACAGTTCCCCATT | Connection etection | |
| J2-Rv | CATTTTATGTTAGCTGGTGGACTGACGCCA |  |  |
