# Supplementary material for: An efficient and versatile reverse genetics system for bovine coronavirus facilitates antiviral evaluation
Source: Vet Res. 2026 Oct 11;57:180. doi: 10.1186/s13567-026-01851-6 (PMC13654213; doi:10.1186/s13567-026-01851-6)
Supplement: Supplementary file 4 [file 13567_2026_1851_MOESM6_ESM.docx]

**Additional file 6. Differences between BCoV XJ2023-WT and rBCoV**

| **Nucleotide position** | **Gene** | **Location** | **Nucleotide difference** | **Amino acid difference** |
| --- | --- | --- | --- | --- |
| 1581 | ORF1a (nsp2) | 457 | AC**C**→AC**T** | Synonymous |
| 2886 | ORF1a (nsp3) | 892 | GG**A**→GG**T** | Synonymous |
| 3603 | ORF1a (nsp3) | 1131 | GC**T**→GC**C** | Synonymous |
| 7638 | ORF1a (nsp3) | 2476 | TA**C**→TA**T** | Synonymous |
| 11358 | ORF1a (nsp6) | 3716 | G**C**T→G**T**T | A→V |
| 14273 | ORF1b (nsp12) | 4688 | AA**T**→AA**C** | Synonymous |
| 16688 | ORF1b (nsp13) | 5493 | GG**T**→GG**C** | Synonymous |
| 18704 | ORF1b (nsp14) | 6165 | GG**C**→GG**A** | Synonymous |
| 23062 | HE | 237 | T**C**T→T**T**T | S→F |
| 24177 | S | 179 | C**A**A→ C**G**A | Q→R |
| 24408 | S | 256 | **C**TG→**T**TG | Synonymous |
| 28719 | M | 10 | GT**T**→GT**A** | Synonymous |
| 29409 | N | 6 | GG**T**→GG**C** | Synonymous |
